# Supplementary material for: Smart soft contact lenses for continuous 24-hour monitoring of intraocular pressure in glaucoma care
Source: Nat Commun. 2022 Sep 20;13:5518. doi: 10.1038/s41467-022-33254-4 (PMC9489713; doi:10.1038/s41467-022-33254-4)
Supplement: Supplementary file 1 — Supplementary Information [file 41467_2022_33254_MOESM1_ESM.pdf]

SUPPLEMENTARY INFORMATION

TITLE

Smart soft contact lenses for continuous 24-hour monitoring of intraocular pressure in glaucoma care

AUTHORS

Jinyuan Zhang[1], Kyunghun Kim[1], Ho Joong Kim[2], Dawn Meyer[3], Woohyun Park[4], Seul Ah Lee[1], Yumin Dai[5], Bongjoong Kim[1,6], Haesoo Moon[1], Jay V. Shah[7], Keely E. Harris[8], Brett Collar[9], Kangying Liu[10], Pedro Irazoqui[9], Hyowon Lee[1,11,12], Shin Ae Park[8*], Pete S. Kollbaum[1,3*], Bryan W. Boudouris[2,10,12*], Chi Hwan Lee[1,3,4,5,11,12*]

[1]Weldon School of Biomedical Engineering, Purdue University, West Lafayette, IN, USA. [2]Charles D. Davidson School of Chemical Engineering, Purdue University, West Lafayette, IN, USA. [3]School of Optometry, Indiana University, Bloomington, IN, USA. [4]School of Mechanical Engineering, Purdue University, West Lafayette, IN, USA. [5]School of Materials Engineering, Purdue University, West Lafayette, IN, USA. [6]Department of Mechanical and System Design Engineering, Hongik University, Seoul 04066, Republic of Korea. [7]Elmore Family School of Electrical and Computer Engineering, Purdue University, West Lafayette, IN, USA. [8]Department of Veterinary Clinical Sciences, Purdue University, West Lafayette, IN, USA. [9]Department of Electrical and Computer Engineering, Johns Hopkins University, MD, USA. [10]Department of Chemistry, Purdue University, West Lafayette, IN, USA. [11]Center for Implantable Devices, Purdue University, West Lafayette, IN, USA. [12]Birck Nanotechnology Center, Purdue University, West Lafayette, IN, USA.

These authors contributed equally: Jinyuan Zhang, Kyunghun Kim, Ho Joong Kim.

These authors jointly supervised this work: Shin Ae Park, Pete S. Kollbaum, Bryan W. Boudouris, Chi Hwan Lee. Email: park1222@purdue.edu (S.A.P.); kollbaum@indiana.edu (P.S.K.); boudouris@purdue.edu (B.W.B.); lee2270@purdue.edu (C.H.L.)

This file contains Supplementary Figures 1–11 and Supplementary Tables 1-2.

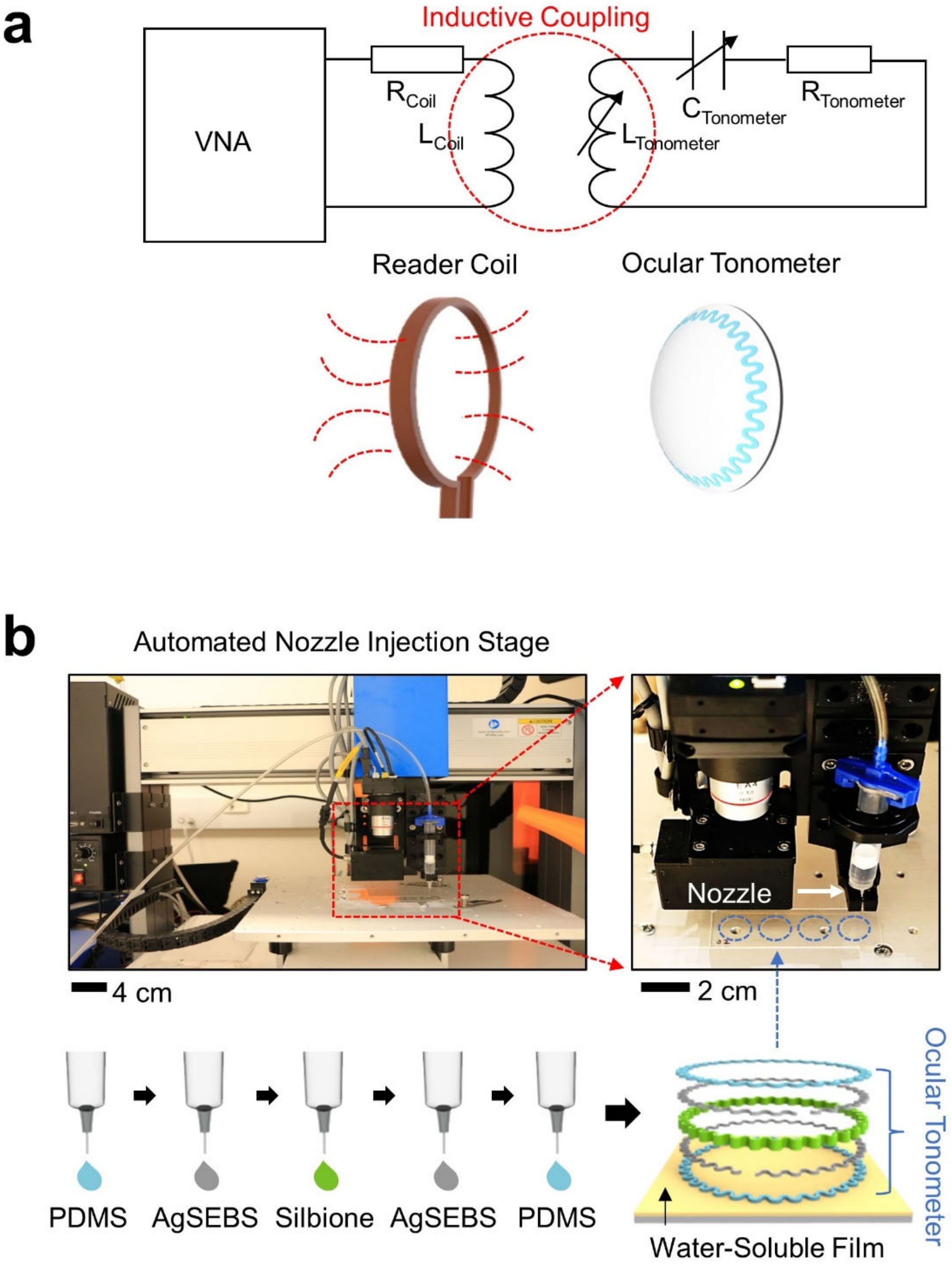

Supplementary Fig. 1. a, Schematic view of the series RLC resonant circuit. **b**, Photographs (top panel) and schematic images (bottom panel) of the automated nozzle injection process.

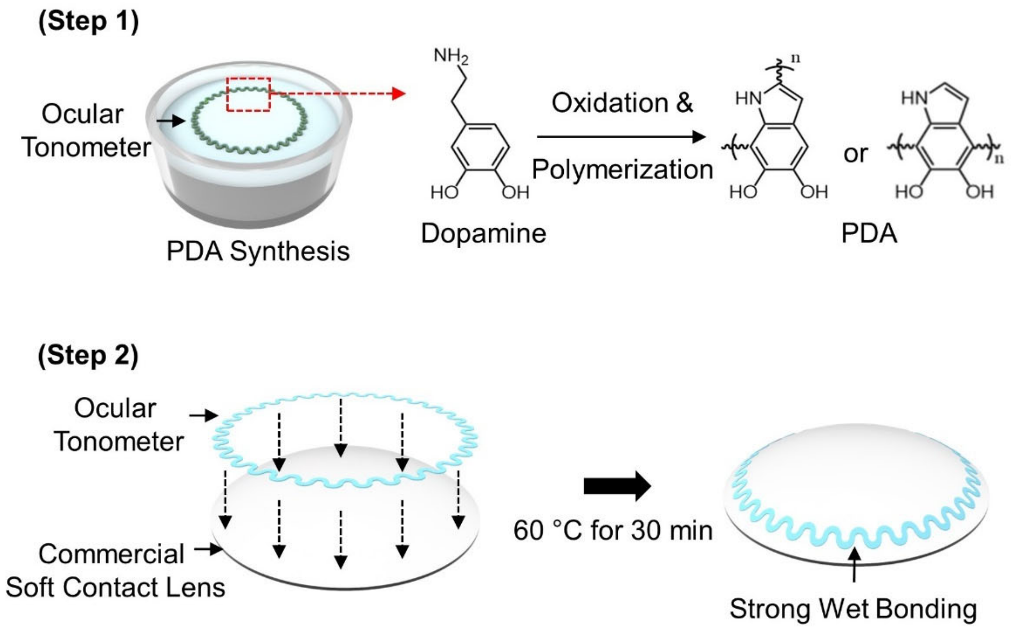

Supplementary Fig. 2. Schematic illustrations for the polymerization of dopamine into a PDA adhesive on the bottom surface of the ocular tonometer (top panel) and the subsequent bonding process onto a commercial soft contact lens.

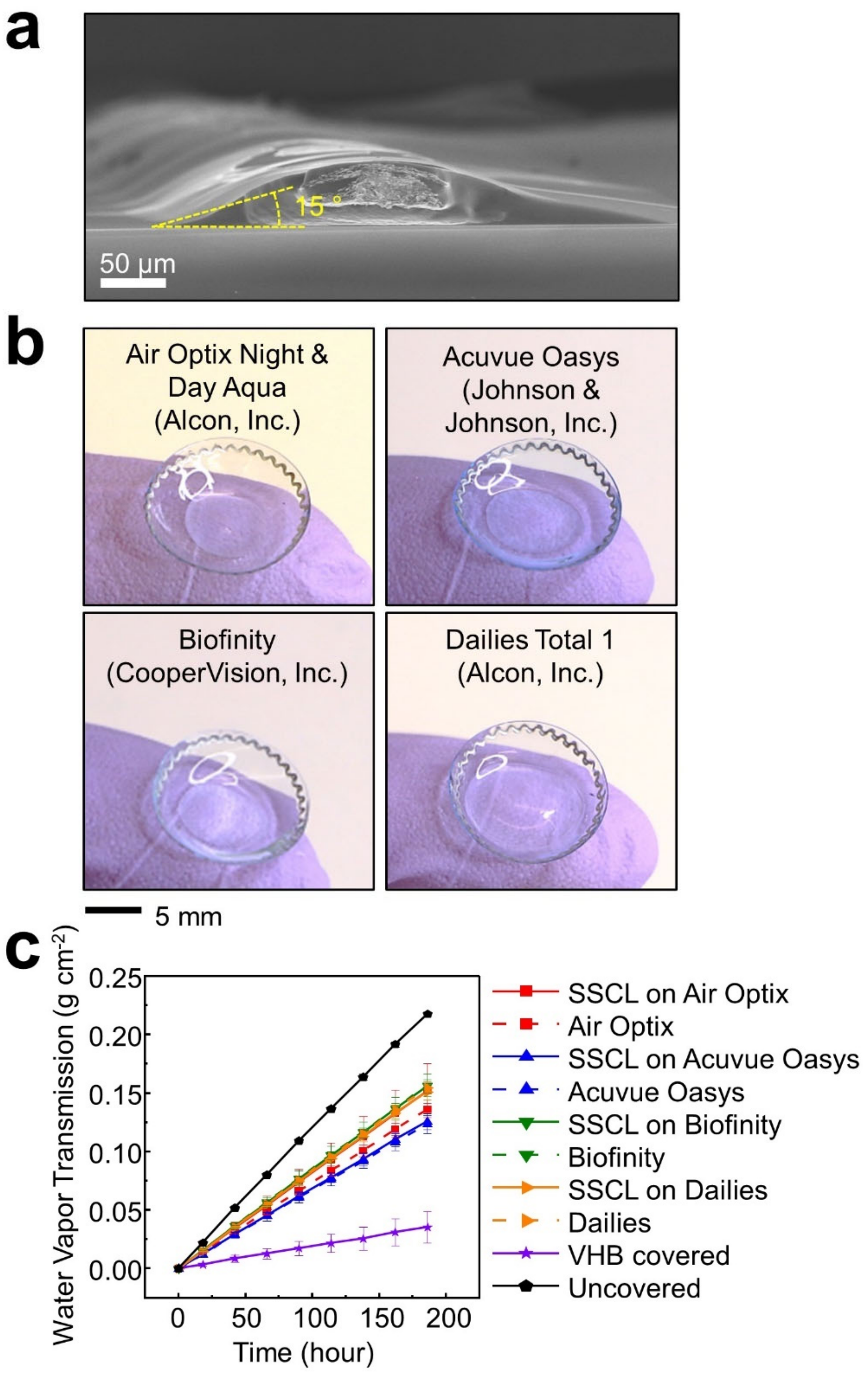

Supplementary Fig. 3. a, Representative cross-sectional SEM image of the internal ocular tonometer. **b,** Photographs of the SSCL built upon various commercial brands of soft contact lenses. **c,** Water vaper transmission of the SSCL upon various commercial brands of soft contact lenses as compared to their bare soft contact lenses (*n* = 3).

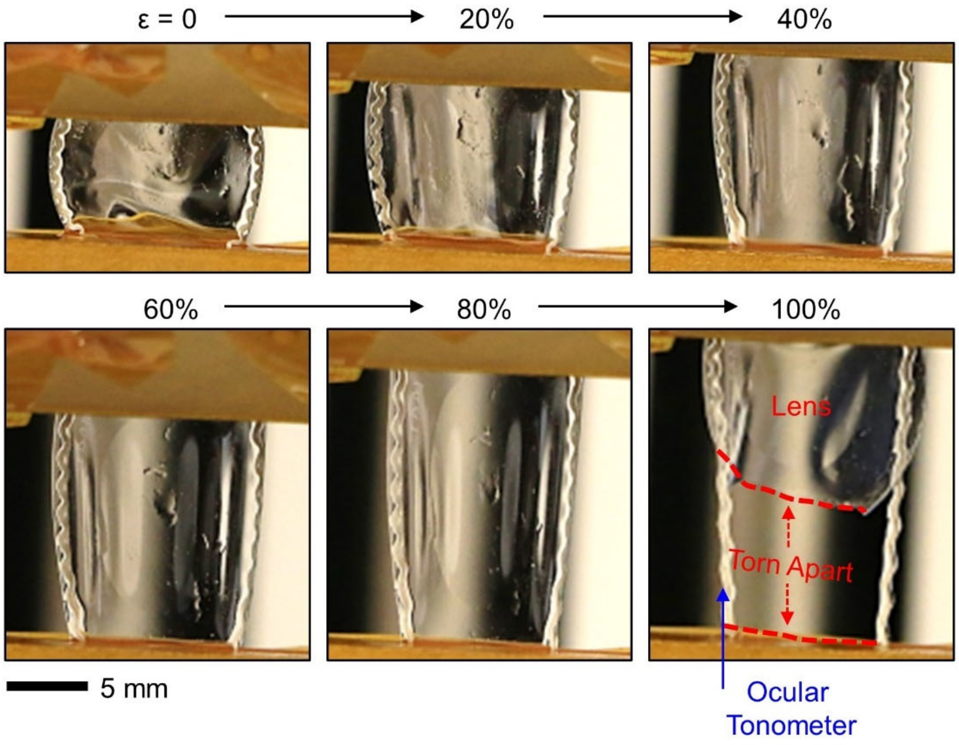

Supplementary Fig. 4. Photographs of the SSCL under stretching up to 100%.

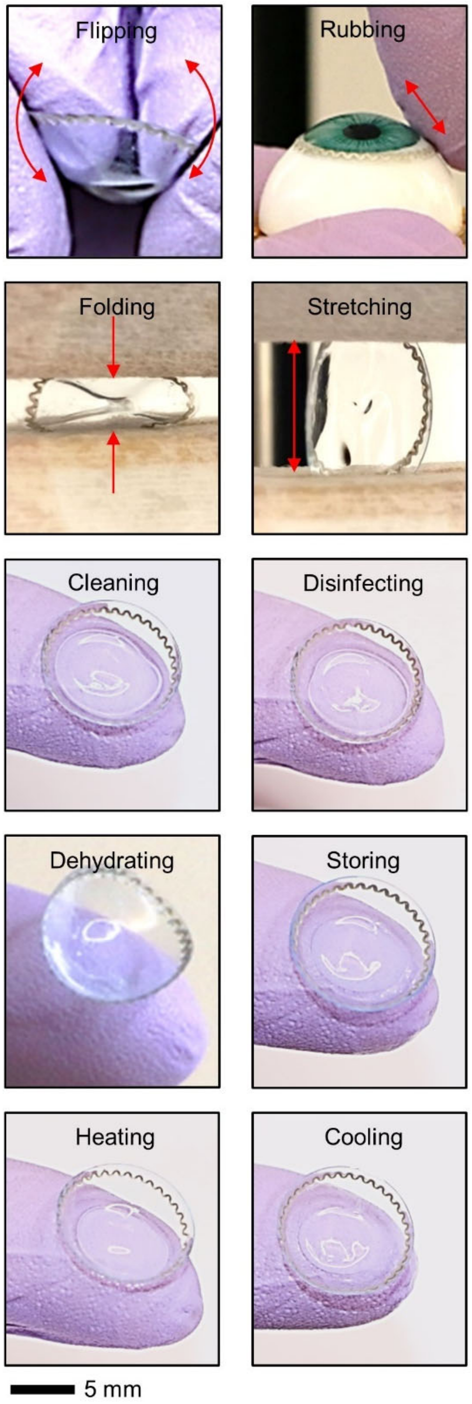

Supplementary Fig. 5. Photographs of the SSCL upon or after flipping, rubbing, folding, stretching, cleaning, disinfecting, dehydrating, storing, heating, and cooling.

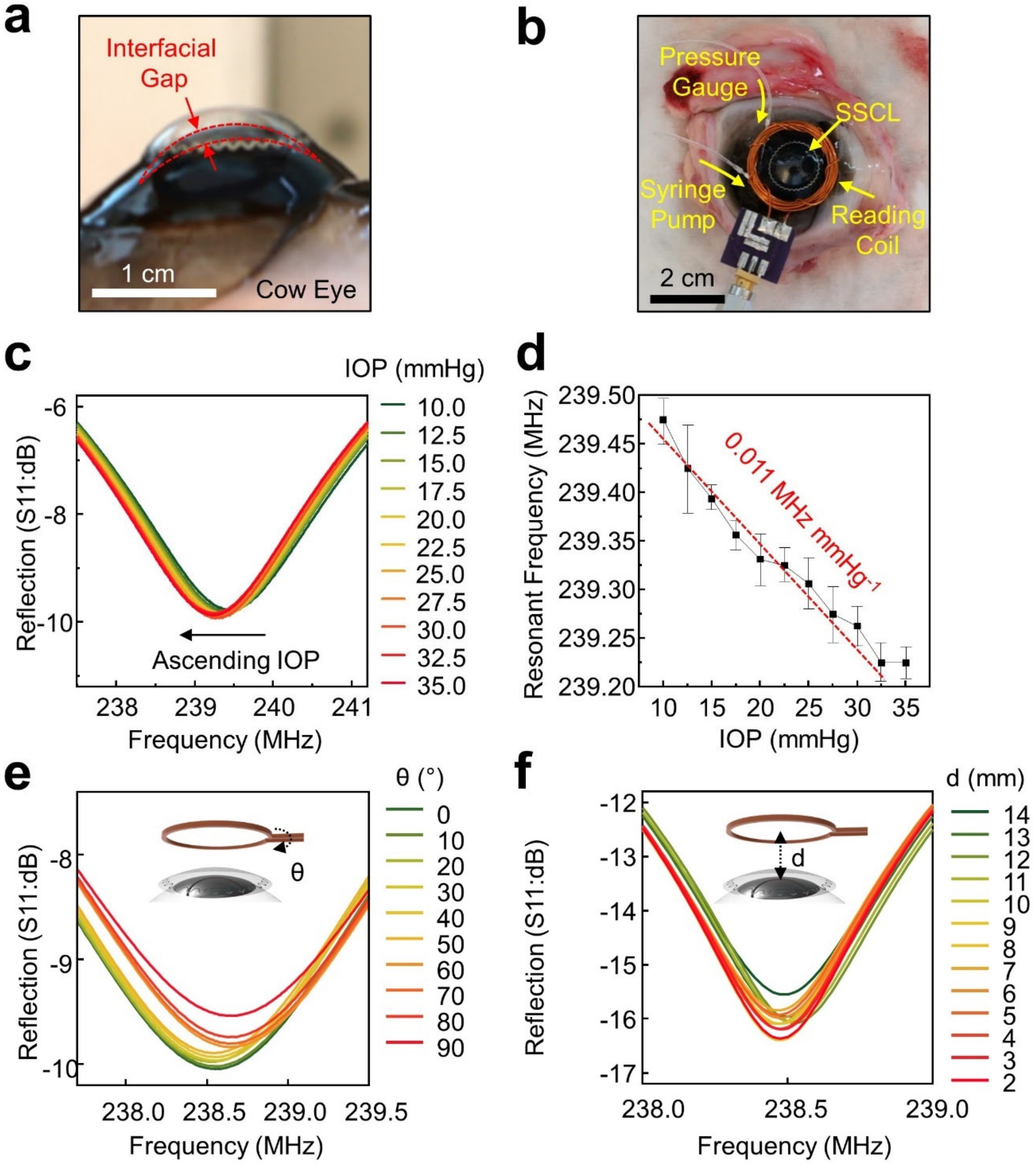

Supplementary Fig. 6. a, Photograph of the SSCL in an enucleated cow eye. **b**, Photograph of the SSCL during IOP monitoring. **c**, Reflection spectra (S11) of the SSCL in response to ascending IOP of the cow eye. **d**, Resonant frequency of the SSCL in response to the IOP of the cow eye (*n* = 3). **e,** Reflection spectra (S11) of the SSCL at various angles with respect to a reader coil. **f,** Reflection spectra (S11) of the SSCL at various distances with respect to a reader coil.

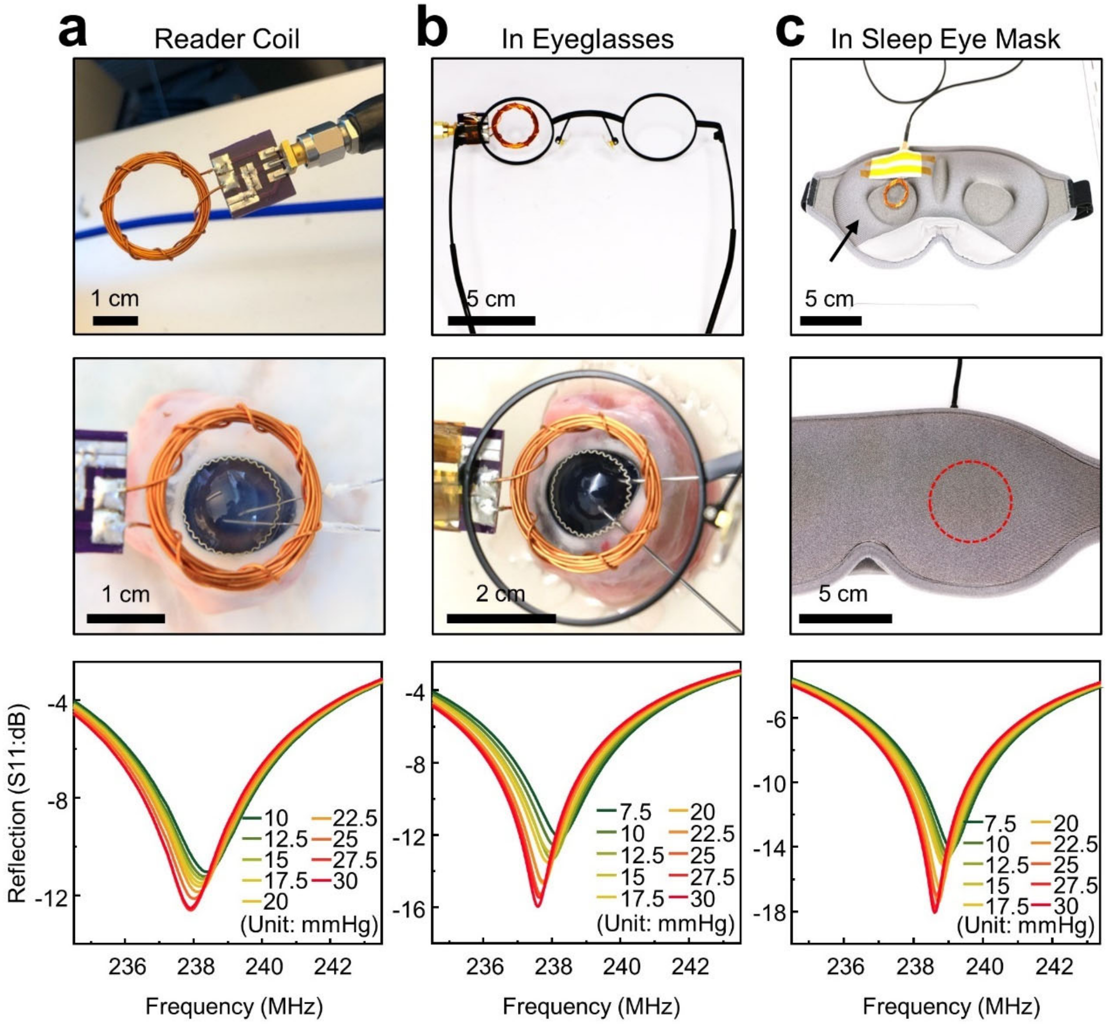

Supplementary Fig. 7. Photographs (top two rows) and reflection spectra (S11) (bottom row) of the SSCL using **(a)** a reader coil as compared to the reader coil embedded within **(b)** eyeglasses and **(c)** a sleep eye mask.

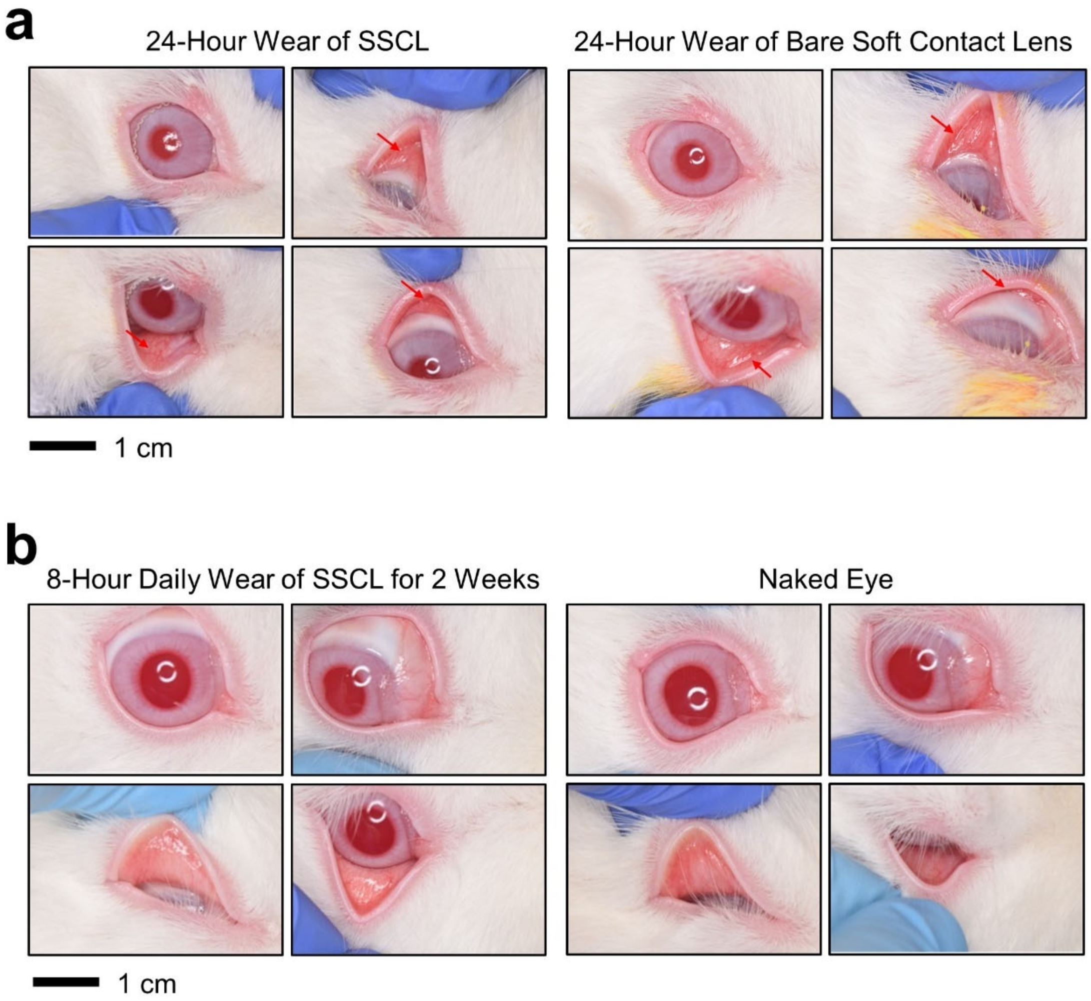

Supplementary Fig. 8. a, Photographs of rabbit eyes after 24-hour wear of the SSCL (left panel) and its bare soft contact lens (right panel). The red arrows indicate the minimal to mild hyperemia of the palpebral conjunctiva. **b,** Photographs of rabbit eyes after 8-hour daily wear of the SSCL for 2 weeks (left panel) as compared to the naked eye (right panel). No abnormality was observed.

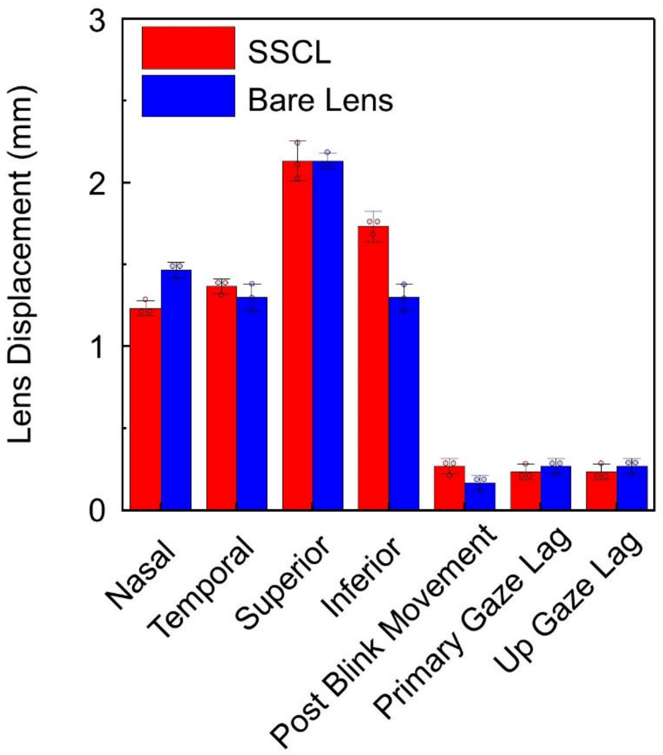

Supplementary Fig. 9. Displacement of the SSCL on a human eye compared to its bare soft contact lens (n = 3).

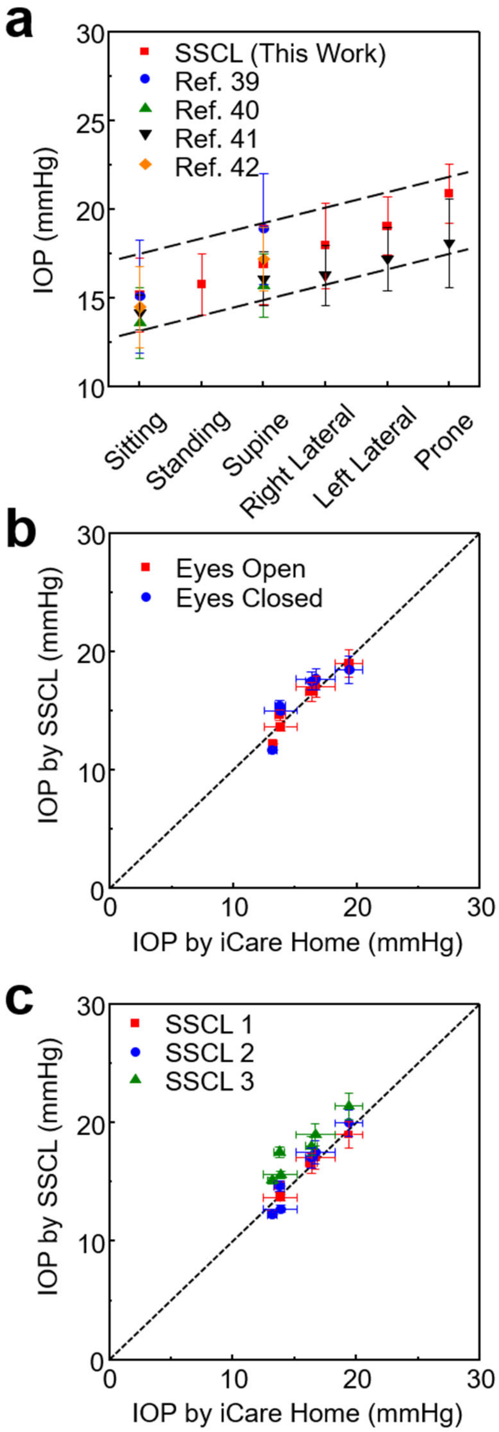

Supplementary Fig. 10. a, Comparison of the IOP data obtained from the SSCL with prior studies. The dashed lines denote the range of IOP under each postural condition in prior studies. **b,** Correlation of the IOP data obtained from the SSCL and the iCare Home with the eye open or closed (n = 3). **c.** Correlation of the IOP data obtained from the SSCL and the iCare Home with different batches of the SSCL (n = 3).

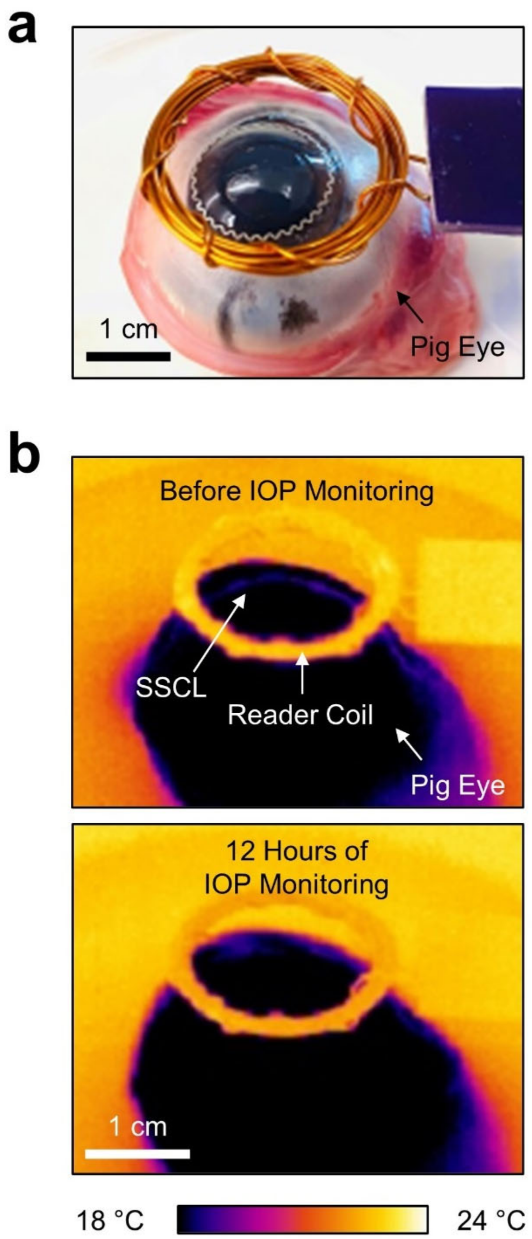

Supplementary Fig. 11. a, Photograph of the SSCL in an enucleated pig eye. **b**, Infrared (IR) image of the SSCL before (top panel) and after (bottom panel) 12 hours of IOP monitoring.

Group I: 24-Hour Continuous Wear		
**Animal ID**	Right Eye – SSCL	Left Eye – Bare Lens Control
**1**	1	2
**2**	2	1
**3**	2	2
**4**	2	1
**Group II: 2-Week Wear (8 Hours Per Day)**		
	Right Eye – SSCL	Left Eye – Untreated Control
**5**	0	1
**6**	1	1

Grades for assessment of inflammation:

0 – None, 1 – Minimal, 2 – Mild, 3 – Moderate, and 4 – Severe.

Supplementary Table 1. Quantitative assessment of histopathologic inflammation grades of the rabbit cornea and conjunctiva.

Validated in Human Eyes	Sensing Materials	Working Principle	Responsivity	Sensitivity	Ref.
Yes	AgSEBS/ Silbione / PDMS	Capacitive / Inductive	0.27 MHz $mmHg^{-1}$	1,121 ppm $mmHg^{-1}$	This Work
Yes	AgNF–AgNW	Piezoresistive	N/A	500 ppm $mmHg^{-1}$	[34]
No	GNWs	Piezoresistive	1.014 kOhm $mmHg^{-1}$	42,250 ppm $mmHg^{-1}$	[35]
No	Graphene / AgNWs	Capacitive / Inductive	2.64 MHz $mmHg^{-1}$	640 ppm $mmHg^{-1}$	[36]
No	Graphene	Piezoresistive	N/A	68,000 ppm $mmHg^{-1}$	[37]
No	Parylene C, Au, PDMS	Inductive	0.0468 MHz $mmHg^{-1}$	111 ppm $mmHg^{-1}$	[38]

Supplementary Table 2. Comparison of the SSCL with current wearable ocular tonometers in terms of sensing materials, working principle, responsivity, and sensitivity.
